# Comparative Investigation of Frankincense Nutraceuticals: Correlation of Boswellic and Lupeolic Acid Contents with Cytokine Release Inhibition and Toxicity against Triple-Negative Breast Cancer Cells

**DOI:** 10.3390/nu11102341

**Published:** 2019-10-02

**Authors:** Michael Schmiech, Sophia J. Lang, Judith Ulrich, Katharina Werner, Luay J. Rashan, Tatiana Syrovets, Thomas Simmet

**Affiliations:** 1 Institute of Pharmacology of Natural Products and Clinical Pharmacology, Ulm University, 89081 Ulm, Germany; michael.schmiech@uni-ulm.de (M.S.); sophia.lang@uni-ulm.de (S.J.L.); judith.ulrich@uni-ulm.de (J.U.); katharina.werner@uni-ulm.de (K.W.); 2 Medicinal Plants Division, Research Center, Dhofar University, Salalah 211, Oman; lrashan@du.edu.om

**Keywords:** frankincense, *Boswellia*, boswellic acid, lupeolic acid, AKBA, cytokine, breast cancer, pentacyclic triterpenic acid, triterpenoid, chorioallantoic membrane assay

## Abstract

For centuries, frankincense extracts have been commonly used in traditional medicine, and more recently, in complementary medicine. Therefore, frankincense constituents such as boswellic and lupeolic acids are of considerable therapeutic interest. Sixteen frankincense nutraceuticals were characterized by high-performance liquid chromatography with tandem mass spectrometry (HPLC-MS/MS), revealing major differences in boswellic and lupeolic acid compositions and total contents, which varied from 0.4% to 35.7%. Frankincense nutraceuticals significantly inhibited the release of proinflammatory cytokines, such as TNF-α, IL-6, and IL-8, by LPS-stimulated peripheral blood mononuclear cells (PBMC) and whole blood. Moreover, boswellic and lupeolic acid contents correlated with TNF-α, IL-1β, IL-6, IL-8, and IL-10 inhibition. The nutraceuticals also exhibited toxicity against the human triple-negative breast cancer cell lines MDA-MB-231, MDA-MB-453, and CAL-51 in vitro. Nutraceuticals with total contents of boswellic and lupeolic acids >30% were the most active ones against MDA-MB-231 with a half maximal inhibitory concentration (IC_50_) ≤ 7.0 µg/mL. Moreover, a frankincense nutraceutical inhibited tumor growth and induced apoptosis in vivo in breast cancer xenografts grown on the chick chorioallantoic membrane (CAM). Among eight different boswellic and lupeolic acids tested, β-ABA exhibited the highest cytotoxicity against MDA-MB-231 with an IC_50_ = 5.9 µM, inhibited growth of cancer xenografts in vivo, and released proinflammatory cytokines. Its content in nutraceuticals correlated strongly with TNF-α, IL-6, and IL-8 release inhibition.

## 1. Introduction

Frankincense is an oleogum resin from trees of the genus *Boswellia* Roxb. ex Colebr., which belong to the *Burseraceae* family. Herbal preparations from frankincense have been used for centuries in traditional Ayurvedic, African, Arab, and Chinese medicine for the treatment of skin ailments, infectious diseases, and other conditions, which today could be assigned to various chronic inflammatory diseases and cancer [1,2]. Likewise, modern medicine has rediscovered frankincense and its pain-relieving, sedative, anti-inflammatory, antimicrobial effects, and even potential anticancer properties [3,4,5].

**Figure 1:**
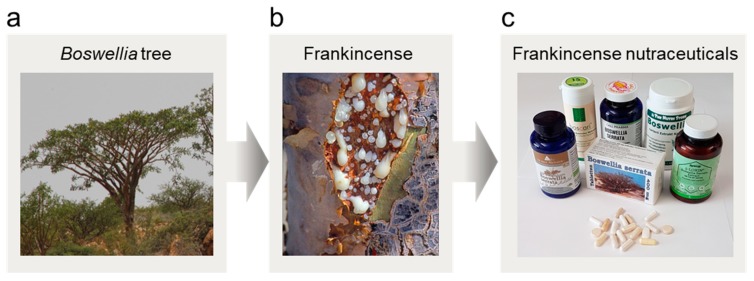
Production of frankincense nutraceuticals. *Boswellia* tree grown in Somalia (**a**), harvesting of the frankincense oleogum resin by bark incisions (**b**), commercial frankincense nutraceuticals (**c**). Pictures with permission from Georg Huber [20].

*Boswellia* trees grow mainly in dry areas in India, the Arabian Peninsula, and the Horn of Africa and often have a shrubby appearance with an average high of 2–6 m [6,7], whereby, the main representative species are *Boswellia sacra* (Oman and Yemen), *Boswellia carterii* (Somalia), and *Boswellia serrata* (India). However, there are about 25 *Boswellia* species reported, but it is still not clear whether this number contains some double-counted species [8]. Frankincense, the oleogum resin of *Boswellia* trees, which contains potential biologically-active compounds, emerges from cuts in trunks and branches as a sticky-milky liquid, which dries quickly in the air, yielding an oleogum resin containing 15–20% boswellic acids and lupeolic acids [9] (Figure 1). These pentacyclic triterpenic acids are believed to be effective in the prevention and treatment of chronic inflammatory diseases and cancer [3,5]. Previous studies have demonstrated that boswellic acids inhibit essential pathways of inflammatory responses by interaction with IκB kinases and therefore inhibition of proinflammatory gene expression or by inhibition of 5-lipoxygenase and leukotrienes biosynthesis [10,11]. Moreover, also recent clinical pilot studies claimed therapeutic efficacy of extracts containing boswellic acids in the treatment of chronic inflammatory diseases like asthma, rheumatoid arthritis, Crohn’s disease, osteoarthritis, collagenous colitis, or colitis ulcerosa [12,13]. Furthermore, *Boswellia serrata* extracts as well as boswellic and lupeolic acids induce apoptosis in various cancer cell lines, such as leukemia, brain, and prostate [5,14,15,16,17]. There have also been reports indicating the efficacy of boswellic acids against breast cancer [18,19].

With 24.2%, breast cancer is the most common cancer of all female cancer cases, causing 15.0% of all female cancer deaths in 2018 [21]. Particularly, triple-negative breast cancer (TNBC) is an aggressive, highly metastatic breast cancer subtype affecting mainly younger women. TNBC is characterized by the lack of expression of hormone receptors (estrogen and progesterone) and the human epidermal growth factor receptor 2 (HER2), which countervails targeted therapy [22]. First, in 2011, Suhail et al. described the induction of apoptosis in human breast cancer cells in vitro by essential oil derived from *Boswellia sacra* [19]. In a later study, we demonstrated that mainly the acidic components of frankincense, i.e., boswellic and lupeolic acids, exhibit cytotoxic efficacy against the TNBC cell line MDA-MB-231 [18].

The global market size of dietary supplements and nutraceuticals was estimated to be worth USD 115 billion in 2018 with an expected compound annual growth rate (CAGR) of 7.8% till 2025 [23]. This demonstrates the strong aptness of the general public to use natural products to maintain good health and to prevent or treat diseases. However, due to insufficient regulation regarding quality and lack of standardization of dietary supplements and nutraceuticals, several questionable or even harmful products are on the market [24].

The aim of this study is to compare frankincense nutraceuticals (FNs) regarding their chemical composition, their efficacies in inhibiting production of inflammatory cytokines, and their cytotoxic efficacy against metastatic TNBC cells.

## 2. Materials and Methods

### 2.1. Materials

All solvents and chemicals were of analytical reagent grade. Dimethyl sulfoxide (DMSO) for sample preparation was purchased from Invitrogen (Thermo Fisher Scientific, Waltham, MA, USA). The solvents used for HPLC-MS/MS analysis were methanol, acetic acid (both HiPerSolv Chromanorm, VWR chemicals, Fontenay-sous-Bois, France) and ultrapure water (reverse-osmosis type water (pureAqua, Schnaitsee, Germany) coupled to a Milli-Q station (Millipore, Eschborn, Germany). The reference substances, acetyl-α-boswellic acid (α-ABA), acetyl-β-boswellic acid (β-ABA), α-boswellic acid (α-BA), β-boswellic acid (β-BA), acetyl-11-keto-β-boswellic acid (AKBA), and 11-keto-β-boswellic acid (KBA) were purchased from Extrasynthese (Genay Cedex, France). Acetyl-lupeolic acid (ALA) and lupeolic acid (LA) were isolated and characterized as previously published [9,25]. Samples of frankincense nutraceuticals (FNs) were purchased from the respective distributors (see Table A1). Stock solutions of FNs and pure boswellic (BAs) and lupeolic acids (LAs) were prepared in DMSO and further diluted in RPMI 1640 medium (Gibco, Thermo Fisher Scientific, Waltham, MA, USA). In all experiments, the final DMSO concentration did not exceed 0.5%.

### 2.2. Quantification of Boswellic and Lupeolic Acids by HPLC-MS/MS Analysis

The method development, chromatographic separation, mass spectrometry detection, and method validation for quantification of boswellic and lupeolic acids by HPLC-MS/MS have been previously published by us [18]. The quantification by HPLC-MS/MS analysis was performed on an Agilent 1260 Infinity system (Agilent, Santa Clara, CA, USA) coupled with an AB API 2000 triple quadrupole mass spectrometer (Applied Biosystems, Foster City, CA, USA). For chromatographic separation, an analytical reversed-phase HPLC column (Dr. Maisch ReproSil-Pur Basic-C18 HD, 3 μm, 125 × 3 mm; Dr. Maisch GmbH, Ammerbruch, Germany) with a precolumn (Dr. Maisch ReproSil Universal RP, 5 μm, 10 × 4 mm) were used. For sample preparation, contents were removed from the capsules, weighted, and dissolved in DMSO (β = 1 mg/mL, *w/v*), whereas pills were beforehand grounded. Samples were filtered through a 0.45 µm regenerated cellulose filter before injection. For quantification, three different capsules or pills were analyzed per product, each in duplicates.

### 2.3. Analysis of Cytokine Release

Whole human blood was collected from the antecubital vein of healthy male donors and incubated with FNs in concentrations of 30 µg/mL for 20 min followed by a stimulation with LPS (10 ng/mL) for 18 h. Plasma was separated by centrifugation and analyzed for IL-10 and TNF-α using ELISA Duo-Set Human from R&D Systems (Minneapolis, MN, USA). Peripheral blood mononuclear cells (PBMC) were isolated from whole venous blood from healthy male donors via density gradient centrifugation using Biocoll (Biochrom GmbH, Berlin, Germany). The collection and analysis of whole blood and peripheral blood mononuclear cells used in this study were approved by the Institutional Ethics Committee (# 177/18). The participating volunteers provided written informed consent to participate in this study. Cells were seeded into a 96-well plate (4 × 10^5^ cells in 200 µL RPMI 1640 supplemented with 1% FCS and 100 U/mL penicillin, 100 µg/mL streptomycin (all from Gibco)). Cells were treated with FNs in concentrations of 10 µg/mL and pure BAs or LAs in final concentrations of 3 µg/mL for 20 min followed by a stimulation with LPS (10 ng/mL) and an 18 h incubation at 37 °C and in a 5% CO_2_ atmosphere. After incubation, cells were centrifuged and supernatants were analyzed. The amounts of IL-12p70, TNF-α, IL-10, IL-6, IL-1β, and IL-8 were quantified by flow cytometry using Cytometric Bead Array (CBA) from Becton Dickinson (Franklin Lakes, NJ, USA) according to manufacturer’s instructions.

### 2.4. Analysis of Antiproliferative and Cytotoxic Effects In Vitro

To verify that the concentrations of the FNs and pure BAs and LAs used, are not cytotoxic to PBMC but cytotoxic to cancer cells, analysis of lactate dehydrogenase (LDH) release (Roche, Basel, Switzerland), XTT assay of cell viability and proliferation (Roche), and analysis of cell membrane integrity by propidium iodide staining [26] were carried out. Triple-negative human breast cancer cells MDA-MB-231 (ATCC, Rockville, MD, USA), CAL-51 and MDA-MB-453 (both from DSMZ, Braunschweig, Germany), and PBMC were analyzed. MDA-MB-231 cells were cultured in Dulbecco’s Modified Eagle Medium (4.5 g/L glucose, GlutaMax; Life Technologies, Carlsbad, CA, USA) supplemented with 10% FCS, 0.1 mM MEM non-essential amino acids, 100 U/mL penicillin, and 100 mg/mL streptomycin. CAL-51 cells were cultured in Dulbecco’s Modified Eagle Medium (4.5 g/L glucose; Life Technologies) supplemented with 10% FCS, 100 U/mL penicillin, and 100 mg/mL streptomycin. The MDA-MB-453 cells were cultured in Leibovitz’s L-15 medium (Life Technologies) containing 10% FCS, 100 U/mL penicillin, and 100 mg/mL streptomycin and kept at atmospheric CO_2_. When cancer cells reached 80% confluence, they were subcultured according to the supplier’s recommendations. MDA-MB-231 and CAL-51 cells were treated 24 h after seeding. MDA-MB-453 cells were allowed to adhere for 72 h, because of slow adhesion. Subsequently, treatment with the respective compounds for 72 h followed. Different concentrations of the respective samples were applied using a Tecan D300e Digital Dispenser (Tecan, Männedorf, Switzerland). Absorbance was measured using an Infinite M1000 PRO Tecan plate reader. For analysis of viability, the blank values containing the respective compounds in the according concentration were subtracted and the percentage of viable cell was calculated by normalization to the vehicle control.

### 2.5. Human Tumor Xenografts on the Chick Chorioallantoic Membrane

TNBC xenografts were established by seeding 0.7 × 10^6^ MDA-MB-231 cells in medium/matrigel (1:1) onto the chick chorioallantoic membrane (CAM) of fertilized chick eggs 7 days after fertilization. For the next 3 consecutive days, cells were treated topically with 20 µL of the respective FN or pure compounds dissolved in 0.9% NaCl (vehicle control: 0.5% DMSO). FN16 was used in concentrations of 5 and 10 µg/mL; AKBA and β-ABA were used in concentrations of 5 µg/mL (10 µM); and doxorubicin was used in a concentration of 1 µM. On day 4 after treatment initiation, tumors were collected, imaged, fixed, and embedded in paraffin for analysis by immunohistochemistry. Tumor volume (mm^3^) was calculated with the formula length (mm) × width^2^ (mm) × π/6. For immunohistochemical analysis, 5-µm slices of the collected tumors were stained using antibodies against the proliferation marker Ki-67. For analysis of apoptosis in vivo, DNA strand breaks were visualized by deoxynucleotidyl transferase dUTP nick end labeling (TUNEL) according to the manufacturer’s recommendations (Roche). Images were recorded with an Axio Lab.A1 microscope (Carl Zeiss, Göttingen, Germany) and a Zeiss 2/3″ CMOS camera using Progres Gryphax software (Carl Zeiss).

### 2.6. Statistical Analysis

Each experiment was repeated at least three times and the data are expressed as mean ± standard deviation (SD) or standard error of the mean (SEM) as indicated. Statistical analysis was performed using Minitab 18 software (Minitab, Munich, Germany) and SigmaPlot 14.0 (Systat Software Inc., San Jose, CA, USA). All data were tested for normal distribution by the Anderson-Darling test and equality of variances by Levene’s test. Sample groups were compared by one-way analysis of variance (ANOVA) and post-hoc by Dunett’s test for parametric data. Non-parametric data were either transformed prior to analysis by Box-Cox transformation or compared directly by Kruskal-Wallis one-way ANOVA on ranks and post-hoc by Dunn’s test. Spearman’s rank correlation was used to investigate correlations of non-parametric data. Cluster analysis was performed with hierarchical agglomerative clustering, complete-linkage, and Euclidean distances. Principal component analysis (PCA) was derived from a covariance matrix of the data.

## 3. Results

### 3.1. Compositions of Boswellic and Lupeolic Acids in Frankincense Nutraceuticals

**Figure 2:**
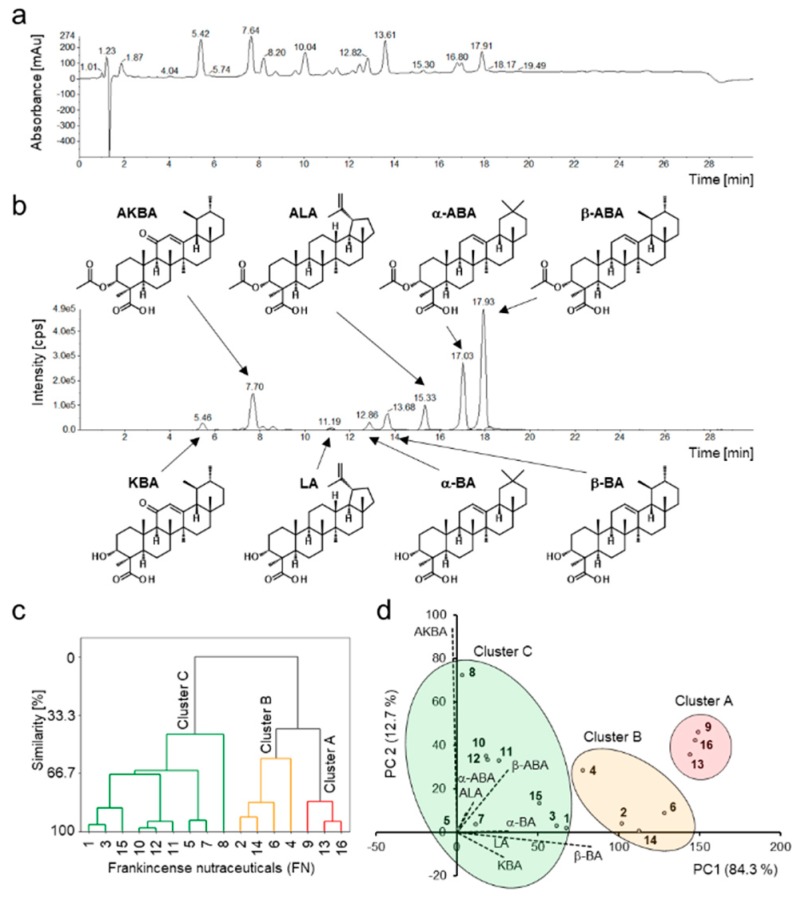
Chromatograms of HPLC-DAD-MS/MS analysis of sample FN9 and multivariate statistical analysis of BAs and LAs contents in different FNs. (**a**) Total wavelength chromatogram with detection at 210 nm, 254 nm, and 280 nm. (**b**) Multiple-reaction monitoring chromatogram and structures of BAs and LAs present in FNs. (**c**) The dendrogram shows FNs assigned to three different clusters according to their BAs and LAs composition: Cluster A >30%, Cluster B with 15–30%, and Cluster C <15% of total BAs and LAs. (**d**) Samples within clusters exhibit similarity in their individual BAs and LAs compositions. Biplot of principal component analysis is shown. Sample FN8 shows the highest deviation due to its unusually high AKBA content concomitantly with very low levels of all other BAs and LAs. Numbered scores visualize the individual FN1-16 in the biplot and dashed lines demonstrating the loadings of the individual BAs and LAs.

Quantification of eight BAs and LAs including α-boswellic acid (α-BA), acetyl-α-boswellic acid (α-ABA), β-boswellic acid (β-BA), acetyl-β-boswellic acid (β-ABA), 11-keto-β-boswellic acid (KBA), acetyl-11-keto-β-boswellic acid (AKBA), lupeolic acid (LA), and acetyl-lupeolic acid (ALA), in 16 frankincense nutraceuticals (FNs) was performed by HPLC-MS/MS. The total contents (*w/w*) of BAs and LAs varied from 0.4% to 35.7% (Table 1). Related to the mass of a capsule or a pill, the total BA and LA contents varied from 3.5 to 157.3 mg/unit (Table S1). Many manufacturers and distributers promote their products as those with boswellic acids contents over 80%. Interestingly, these concentrations could not be confirmed. Manufacturers usually determine the boswellic acid content of their products by non-aqueous titration, for example, using 0.1 N potassium methoxide and 0.3% (*w/v*) thymol blue as the indicator [27]. These methods estimate unselectively all acidic compounds, not only boswellic acids. Here, a highly selective and accurate quantification method for analysis of BAs and LAs by HPLC-MS/MS was applied (Figure 2a,b). Consequently, the precise contents of the individual BAs and LAs in nutraceuticals could be determined (Table 1 and Table S1).

In a previous study, we showed that oleogum resins of the species *B. serrata* are characterized by higher proportions of deacetylated compounds compared to acetylated compounds [18]. This pattern was also observed in the FN samples analyzed here, which contained mainly extracts from *B. serrata*. Unusually, particularly for *B. serrata*, sample FN8 exhibited an exceptionally high content of AKBA. Especially, the concurrence of the high content of AKBA and very low contents of the other BAs and LAs indicated a special enrichment procedure putting FN8 apart from the other samples. Meins et al. observed the same uncommon ratio between AKBA content and total BAs content in a product containing the same 5-Loxin^®^ extract [28]. FN5 and FN7 showed the lowest contents of BAs and LAs of all investigated samples, with total BAs and LAs contents of 2.9% for FN5 and only 0.9% for FN7. In addition to the exceptionally low BAs and LAs contents, the large difference between the stated extract content of 400 mg for FN7 and the determined pill mass of 809.8 ± 5.0 mg points also to an oddly high amount of additives in FN7 (Table 1 and Table S1). According to the manufacturer, the AKBA content of FN7 is 52 mg per pill. However, analysis revealed an AKBA content <1 mg per pill. Hence, it can be assumed that either less *Boswellia* extract than stated was processed or other *Boswellia* oleogum resins containing little or no BAs and LAs were used like, for example, *B. frereana* [18,29].

Multivariate statistical methods, cluster analysis and principal component analysis (PCA) were applied to the datasets to visualize the data and discover patterns of the BAs and LAs compositions in different FNs. Cluster analysis assigned the samples to three different clusters (Figure 2c). Cluster A contained samples FN9, FN13, and FN16 with the highest total BAs and LAs contents of over 30%. Furthermore, these FNs showed high similarity regarding the composition of the individual components (Figure 2d). Cluster B (samples FN2, FN4, FN6, and FN14) comprised samples with total BAs and LAs contents between 15% and 30%, and Cluster C (samples FN1, FN3, FN4, FN5, FN7, FN8, FN10, FN11, and FN12) contained less than 15% BAs and LAs. FN8 showed the lowest similarity to Cluster C (<50%) due to its unusual chemical composition with high AKBA concentration. Moreover, samples FN10, FN11, and FN12, which contained non-extracted oleogum resin, formed an additional subcluster, due to their similarity. Interestingly, these three samples contained oleogum resins of different *Boswellia* species. According to the manufacturers, sample FN10 was made of *B. sacra*, FN11 of *B. serrata*, and FN12 of *B. carterii*. Comparing with the BAs and LAs compositions determined in our previous studies [9,18], FN11 exhibited indeed general BAs and LAs patterns typical for *B. serrata* resins, with a higher proportion of deacetylated BAs and LAs than acetylated ones. However, FN10 and FN12 showed BAs and LAs patterns untypical for *B. sacra* or *B. carterii* resins, but more similar to *B. serrata*, too. Because these samples contain no extracts but untreated oleogum resin, these differences could not be caused by an extraction procedure. Therefore, it cannot be excluded that instead of *B. sacra* or *B. carterii*, possibly *B. serrata* oleogum resin might have been used for manufacturing of FN10 and FN12.

### 3.2. Inhibition of Proinflammatory Cytokine Release by Frankincense Nutraceuticals

**Figure 3:**
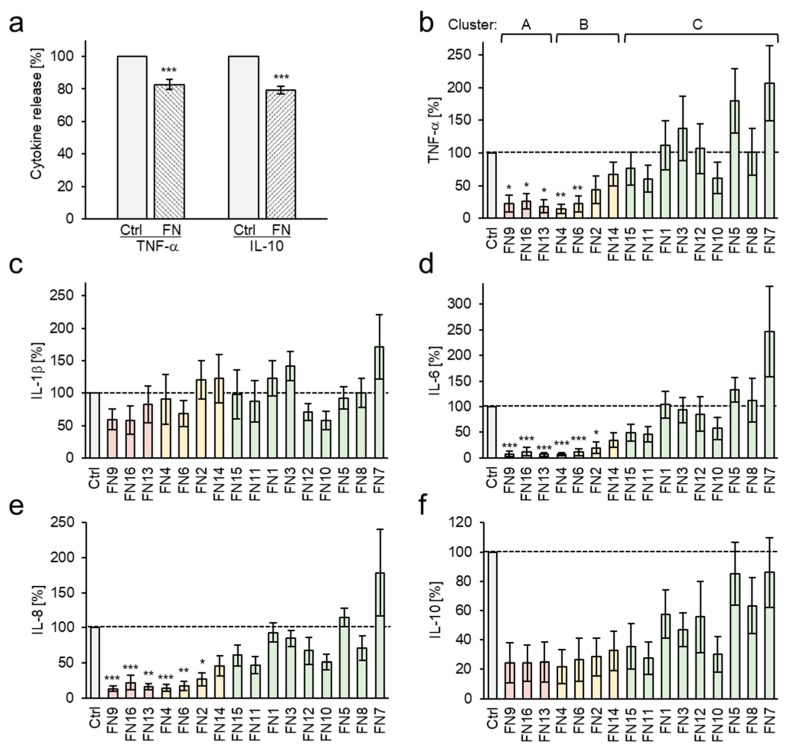
FNs inhibit cytokine production by LPS-stimulated blood and PBMC. (**a**) Inhibition of TNF-α and IL-10 expression by FNs (each at 30 µg/mL) in LPS-stimulated whole blood. Data are mean ± SEM of 16 FNs; each FN was analyzed in five independent experiments/donors, Wilcoxon test. (**b**–**f**) Inhibition of TNF-α, IL-1β, IL-6, IL-8, and IL-10 production by LPS-stimulated PBMC by the respective FN (each at 10 mg/mL). Samples are arranged according to β-ABA content in a descending order. Clusters are from cluster analysis of BAs and LAs compositions in Figure 2c,d: Cluster A (light red) >30%, Cluster B 30–15% (yellow), and Cluster C (green) <15% total contents of BAs and LAs. Box-Cox transformation followed by one-way ANOVA and post-hoc Dunnett’s test. All data are mean ± SEM. *n* = 7 donors; * *p* < 0.05, ** *p* < 0.01, *** *p* < 0.001 compared to LPS-stimulated control groups (Ctrl).

For comparative investigation of the immunomodulatory activities of the FNs, whole blood of healthy donors was treated with lipopolysaccharide (LPS) to initiate an acute inflammatory response and to induce the release of proinflammatory cytokines. Pretreatment with FNs significantly decreased the expression of the proinflammatory cytokine TNF-α compared to the LPS-treated control group (Figure 3a). Interestingly, the production of the anti-inflammatory cytokine IL-10 was inhibited by the FNs, too. IL-10 acts to limit inflammatory responses by damping the uncontrolled production of proinflammatory cytokines including TNF-α [30]. However, also TNF-α regulates the production of IL-10. Thereby, the chronological sequence of cytokine expression during inflammation is essential. While high levels of proinflammatory cytokines IL-1, IL-6, IL-8, and TNF-α are released already 4–8 h after LPS stimulation, maximal levels of the anti-inflammatory IL-10 are observed only 24–48 h after stimulation [31]. Moreover, about 50–75% of IL-10 released by LPS-stimulated monocytes can be inhibited by anti-TNF-α [32], indicating that TNF-α is responsible for the majority of the released IL-10. Therefore, the decreased production of TNF-α by blood treated with FNs would decrease the later IL-10 release (Figure 3a).

The strong albumin-binding affinity of BAs complicates an accurate analysis of their immunomodulatory activity in whole blood [33]. In addition, blood contains varying amounts of different lipids aiding samples’ solubility. Hence, analysis of variance (ANOVA) exhibited no significant differences between the samples or sample groups when analyzed in whole blood. As monocytes are the major producers of TNF-α and IL-10 in blood [34], for further analysis, peripheral blood mononuclear cells (PMBC) were used and the effects of the FNs on the production of a panel of cytokines including TNF-α, IL-1β, IL-6, IL-8, IL-10, and IL-12p70, were analyzed. Contents of IL-12p70 were too low for an adequate evaluation and are not presented. FNs were used at a concentration of 10 µg/mL, which did not affect cell viability as analyzed by XTT and lactate dehydrogenase release (LDH) assays and by propidium iodide staining.

FNs with BAs and LAs contents >30% (Cluster A: FN9, FN13, and FN16) exhibited a significant inhibition of TNF-α, IL-6, and IL-8 expression compared to the control LPS group (Figure 3b–f and Table S2). Moreover, FN4 and FN6 from Cluster B showed considerable inhibitory activity towards TNF-α, IL-6, and IL-8, too. The most potent five nutraceuticals, FN4, FN6, FN9, FN13, and FN16, decreased the release of proinflammatory cytokines to 20.7% for TNF-α, 9.2% for IL-6, and 16.8% for IL-8 (average values for all five FNs). Interestingly, these five samples also had the highest concentrations of ALA, α-ABA, and β-ABA. The proteasome inhibitor MG132 used as a positive control decreased cytokine release to 15.5% ± 7.2% for TNF-α, 18.5% ± 7.7% for IL-1β, 5.2% ± 3.5% for IL-6, 20.2 ± 7.2% for IL-8, and 37.5% ± 14.8% for IL-10 compared to the control group. Differently, samples FN5 and FN7, that were previously shown to contain the lowest BAs and LAs contents, exhibited no inhibitory effects on cytokine production at all but even enhanced the expression of the proinflammatory cytokines TNF-α, IL-6, and IL-8 with average values of 142.4% for FN5 and 210.7% for FN7 compared to the control group. The reason for the increased secretion of cytokines might be contamination of these FNs with bacterial products.

Investigation of the correlation between the chemical composition and cytokine expression revealed that the total amount of BAs and LAs correlate positively with the inhibition of TNF-α, IL-6, IL-8, and IL-10 (Table 2 and Figure S1). Especially, the acetylated compounds ALA, α-ABA, and β-ABA exhibit the highest correlation coefficients, while the acetylated compound with keto group AKBA shows the lowest correlation. However, when FN8 with the extraordinarily high AKBA content was removed as an outlier (Grubbs test), AKBA contents in FNs exhibited significant correlation with all cytokines with *p* = 0.007 for TNF-α, *p* < 0.001 for IL-1β, *p* = 0.011 for IL-6, *p* = 0.006 for IL-8, and *p* = 0.001 for IL-10. Inhibition of IL-1β correlated only with AKBA, ALA, and α-ABA contents in FNs. Moreover, the inhibition of all five investigated cytokines correlated with each other. Whereby, IL-1β, the only cytokine in the panel activated by the NLRP3 inflammasome [35], showed the lowest correlation (Table 2 and Figure S1).

### 3.3. Cytotoxic Efficacy of Frankincense Nutraceuticals Against Triple-Negative Breast Cancer Cells In Vitro

**Figure 4:**
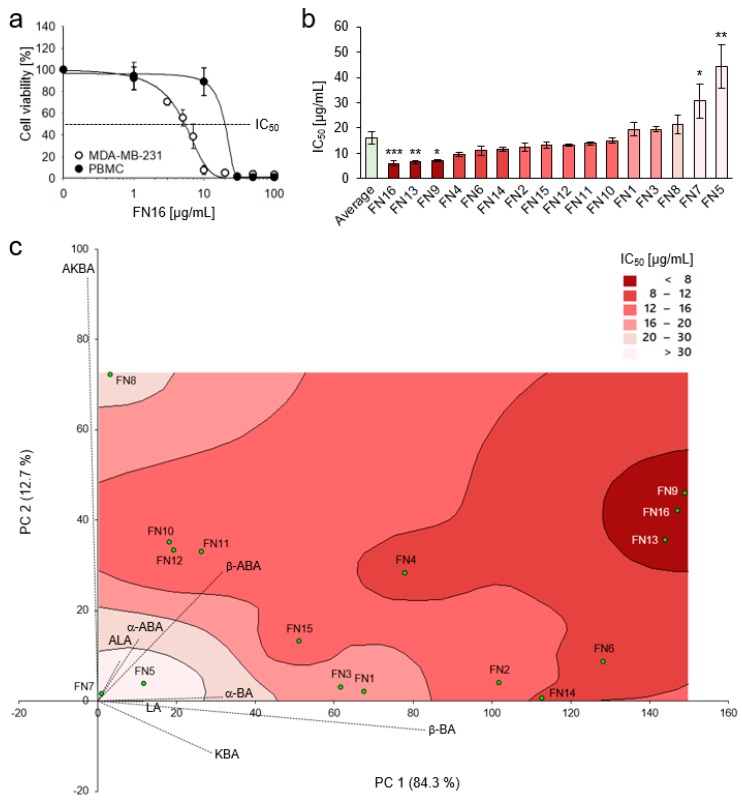
FNs are toxic to a TNBC cell line. (**a**) MDA-MB-231 cancer cells and peripheral blood mononuclear cells (PBMC) were treated for 72 h with FN16, and cell viability was analyzed by XTT assay (*n* = 3). (**b**) Half maximal inhibitory concentrations (IC_50_) of FNs against MDA-MB-231 cell line. Comparison of IC_50_ values of individual FNs with the average IC_50_ value for all FNs (15.9 µg/mL) by Box-Cox transformation, one-way ANOVA, and post-hoc Dunnett’s test. All data are mean ± SEM, *n* = 3 for the respective FN, *n* = 16 for the average value, * *p* < 0.05, ** *p* < 0.01, and *** *p* < 0.001. (**c**) Principal component analysis (PCA) and contour plot visualizing the correlation between toxicity of different FNs and their BAs and LAs contents.

FN16, one of the most potent nutraceuticals with respect to cytokine release, concentration-dependently inhibited the viability of the highly metastatic, treatment-resistant breast cancer cell line MDA-MB-231 in vitro. Interestingly, cancer cells were more sensitive to FN16 compared to normal human PBMC, providing evidence for selectivity against cancer cells (Figure 4a). Also, FN1 with low contents of BAs and LAs, exhibited higher toxicity against MDA-MB-231 cells compared to PBMCs (Figure S2a), though the difference was not as strong as for FN16. Therefore, all FNs were investigated for their toxicity against MDA-MB-231 breast cancer cells and the half maximal inhibitory concentrations (IC_50_) were determined (Table 3 and Figure 4b). The FNs exhibited cytotoxicity against MDA-MB-231 cells with an average IC_50_ of 15.9 µg/mL. The samples FN9, FN13, and FN16 (Cluster A) with the highest BAs and LAs contents exhibited also significantly higher cytotoxicity with IC_50_ values for FN9, FN13, and FN16 of 7.0 µg/mL, 6.6 µg/mL, and 6.0 µg/mL, respectively, compared to the average IC_50_ value of 15.9 µg/mL (Figure 4b). Samples FN5 and FN7 (Cluster C) demonstrated the lowest cytotoxicity against MDA-MB-231 with an IC_50_ = 44.3 µg/mL for FN5 and an IC_50_ = 30.7 µg/mL for FN7. Thus, FN5 and FN7 not only exhibited the lowest contents of BAs and LAs and the poorest cytokine modulatory properties, but also the cytotoxic efficacies against MDA-MB-231 were significantly below average. Hence, this points to a correlation between BAs and LAs compositions and cytotoxicity against breast cancer cells. A combination of principal component analysis (PCA) of the BAs and LAs concentrations with a contour plot that visualizes the IC_50_ values revealed correlation between the BAs and LAs contents and cytotoxic efficacy (Figure 4c). Interestingly, in addition to similar total BAs and LAs contents and similar effects on cytokine release, the compositions of the individual BAs and LAs in the most potent nutraceuticals FN9, FN13, and FN16 were highly alike. Spearman’s rank correlation analysis revealed that contents of BAs and LAs without a keto group exhibit the highest correlation to FN cytotoxic efficacy (Figure S1). In this regard, the content of the boswellic acid β-ABA shows the highest correlation to cytotoxicity against MDA-MB-231.

Furthermore, a principal component regression analysis (PCR) based on known BAs and LAs contents in FNs was used, to avoid biases caused by multicollinearity. Sample FN5 was identified as an outlier by the Grubbs test and excluded from the regression analysis. The resulting regression model exhibited a *R*^2^ of 96.5% and a significance of correlation of *p* < 0.001. The regression analysis yielded the following equations:

(1) $${\mathrm{IC}}_{50}\left(\mu g/\mathrm{mL}\right)=31.6-0.223\;PC1-0.668\;PC2+0.000586\;PC1^{2}+0.00737\;PC2^{2}+0.00166\;PC1\times PC2$$

with

(2) PC1=0.289 [KBA]+0.115 [LA]+0.316 [α-BA]+0.830 [β-BA]−0.026 [AKBA]+0.056 [ALA]+0.103 [α-ABA]+0.316 [β-ABA]

and

(3) PC2=−0.115 [KBA]−0.002 [LA]+0.008 [α-BA]−0.064 [β-BA]+0.936 [AKBA]+0.088 [ALA]+0.136 [α-ABA]+0.285 [β-ABA]

with concentrations of corresponding BA or LA in µg/mg FN given in square brackets.

The IC_50_ values calculated by the regression model exhibited an average absolute residue of only ± 1.0 µg/mL compared to the experimentally-derived values (Table 3). Hence, the regression equation could be used as a tool to predict cytotoxic efficacy of FNs and other frankincense herbal preparations with known BAs and LAs content. By expanding the data set, one could achieve an even more accurate prediction.

Further, the three FNs, FN9, FN13, and FN16, showing the highest cytotoxicity against MDA-MB-231 cells, were investigated for their cytotoxic efficacy against two additional TNBC cell lines, MDA-MB-453 and Cal-51. Likewise, the investigated samples exhibited considerable cytotoxicity against MDA-MB-453 with IC_50_ values between 12.9 µg/mL and 17.3 µg/mL, and against Cal-51 with even lower IC_50_ values between 3.8 µg/mL and 4.0 µg/mL (Table 4). This indicates that FNs exhibit cytotoxic efficacy against different triple-negative treatment-resistant breast cancer cells.

The non-halogenated anthracycline doxorubicin, a chemotherapeutic agent used to treat patients with breast cancer, was analyzed as a positive control for FNs. Doxorubicin exhibited an IC_50_ = 0.41 ± 0.03 µg/mL for MDA-MB-231, an IC_50_ = 0.26 ± 0.02 µg/mL for MDA-MB-453, and an IC_50_ = 0.033 ± 0.004 µg/mL for Cal-51. However, for doxorubicin, severe adverse effects including cardio- and nephrotoxicity have been reported [36,37]. Differently, for frankincense extracts, only mild adverse effects, like heartburn or nausea, have been described [38].

### 3.4. Boswellic and Lupeolic Acids Inhibit Cytokine Release and Are Cytotoxic for TNBC Cells

Cytotoxicity of FNs against MDA-MB-231 cells correlated significantly to the total contents of BAs and LAs as well as to individual BAs and LAs contents, except for AKBA, in FNs (Spearman’s rank correlation, *p* < 0.001) (Figure S1). The highest correlation coefficient exhibited β-ABA, whereas no significant correlation was detected for AKBA. The poor correlation of AKBA was caused mainly by the unusual chemical composition of the sample FN8 with unexpectedly high AKBA content concomitantly with very low contents of other BAs and LAs. After rejecting sample FN8 from the statistical analysis, the AKBA contents of the remaining samples correlated significantly with the cytotoxicity against MD-MB-231 cells (*p* = 0.004). Hence, we further investigated the cytotoxic efficacies of the pure individual BAs and LAs against MDA-MB-231 (Table 5). The acetylated forms of boswellic acids were more effective against MDA-MB-231 cells than their deacetylated forms; and the most cytotoxic compounds were AKBA with IC_50_ = 6.6 µM, α-ABA with IC_50_ = 7.2 µM, and β-ABA with IC_50_ = 5.9 µM. Differently, for lupeolic acids, LA was more cytotoxic compared to ALA. We have previously shown that acetylated BAs, similar to anticancer drugs such as camptothecins and podophyllotoxins, inhibit activities of human topoisomerases [39]. Acetylated BAs also inhibit the activation of transcriptional factor NF-κB which regulates the synthesis of antiapoptotic proteins [11,17], whereas ALA is an inhibitor of the AKT kinase [16]. These molecular targets might explain the high cytotoxicity of BAs and LAs against proliferating cancer cells.

Interestingly, similar to FN1 and FN16, AKBA, and in particular, β-ABA, showed higher toxicity against cancer cells compared to PBMC (Figure S2b,c), suggesting selectivity against cancer cells.

**Figure 5:**
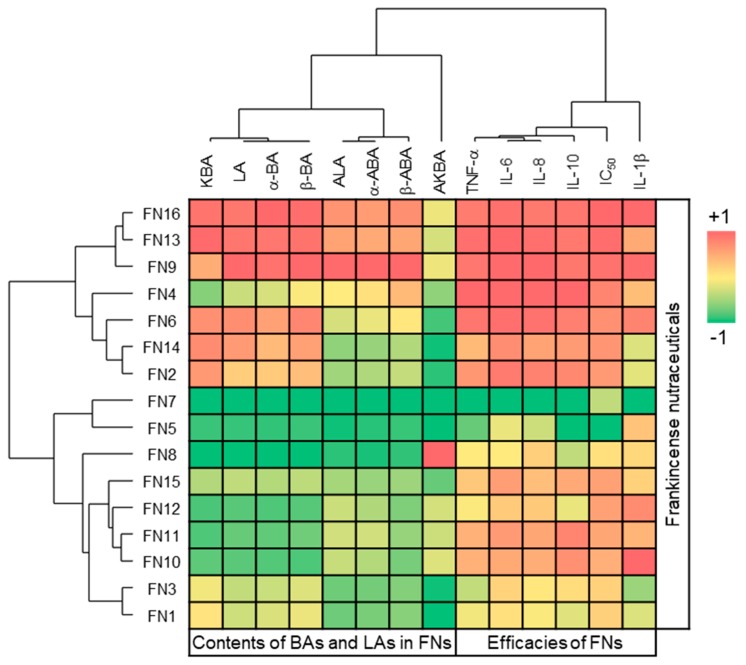
Heatmap visualizing correlations between BAs and LAs compositions in FNs and FN efficacies with respect to inhibition of cytokine release and cancer cell toxicity. Cluster analysis of variables (BAs, LAs, cytokines, IC_50_) was performed with distances of correlation coefficients and complete linkage. Cluster analysis of objects (FN1-16) was performed with standardized variables, Euclidean distances, and complete linkage. All data were standardized and efficacy values were additionally inverted. +1 indicates high contents of BAs and LAs in FNs or high efficacies of FNs, −1 indicates low contents or efficacies. BAs and LAs contents were analyzed by HPLC-MS/MS; IC_50_ toxicity against MDA-MB-231 breast cancer cells was determined by XTT (72 h); and inhibition of cytokine release by PBMC was analyzed after 18 h by flow cytometry.

Moreover, toxicity against breast cancer cells strongly correlated with cytokine inhibition with *p* = 0.023 for IL-1β and *p* < 0.001 for TNF-α, IL-6, IL-8, and IL-10 (Figure S1). Cluster analysis of variables confirmed the correlation between toxicity against breast cancer cells and cytokine inhibition (Figure 5). In addition to the activation of genes coding for antiapoptotic proteins and for those necessary for continuous cell proliferation, NF-κB activation is central to the activation of cytokine and chemokine gene expression [40]. In previous studies, we have shown that acetylated boswellic acids AKBA and β-ABA specifically inhibited the activities of human IκB kinases (IKK) and the subsequent release of proinflammatory cytokines by human monocytes [11]. Moreover, intercepting the IKK activity by AKBA and β-ABA inhibited proliferation and promoted apoptosis in androgen-independent PC-3 prostate cancer cells in vitro and in vivo [17]. Hence, inhibition of NF-κB by boswellic acids might represent a clue as to why these compounds inhibit both inflammatory processes and cancer growth.

Interestingly, chronic inflammation is known to increase cancer risk. Particularly, activation of NF-κB and production of inflammatory cytokines by tumor-associated immune cells are important components aiding tumor initiation, growth, malignant transformation, invasion, and metastasis [41]. Hence, inhibition of inflammatory mediators prevents the development of experimental cancers, and treatment with anti-inflammatory drugs reduces cancer risks and progression [42]. Hence, inhibition of inflammation by frankincense constituents could benefit anticancer therapy.

Similar to FNs, pure BAs and LAs, in concentrations which do not affect PBMC viability, inhibited cytokine production by LPS-activated PBMC, but not as efficiently as FNs (Table S3). Although BAs and LAs are identified here as the active principle of FNs, frankincense extracts also contain a rather complex lipidome including a large number of different fatty acids [43]. The lipids are extracted from frankincense during extract preparation and are persistent components of FNs. Such lipids can act as a natural emulsifier for nonpolar compounds like BAs and LAs and increase their bioavailability [44]. Accordingly, the intake of a FN in combination with a high-fat meal improved the bioavailability of BAs in humans [45]. Hence, the intake of pure BAs and LAs in the absence of solubility enhancers might decrease their bioavailability and worsen the therapeutic response. This should be considered in the development of drugs based on BAs and LAs. Formulations with lecithin for oral administration and complexation with cyclodextrins or liposomes for injections can be beneficial [46,47].

### 3.5. Inhibition of Proliferation and Induction of Apoptosis in Breast Cancer Xenografts

The antitumor activity of FNs and BAs was further verified in vivo, in MDA-MB-231 breast cancer xenografts grown on the chorioallantoic membrane (CAM) of fertilized chick eggs. Xenografts were treated with FN16, because it contains high amounts of BAs and LAs and exhibited the highest cytotoxicity against MDA-MB-231 cells in vitro. Also, two acetylated BAs, AKBA and β-ABA, which exhibited the highest cytotoxic efficacy in vitro, were tested in an in vivo model.

**Figure 6:**
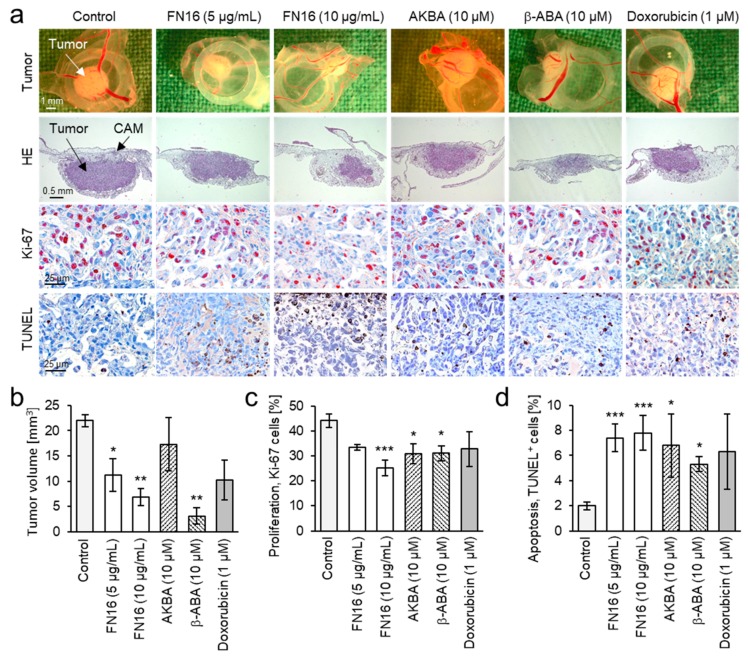
FNs and BAs inhibit growth and induce apoptosis in TNBC breast cancer xenografts in vivo. MDA-MB-231 cells were grafted on the chorioallantoic membrane (CAM) of fertilized chick eggs and treated for 3 consecutive days with either FN16 (5 mg/mL and 10 mg/mL), the boswellic acids AKBA or β-ABA (each 10 µM), doxorubicin (1 µM), or DMSO (0.5%) as control. (**a**) First row: tumor photographs immediately after extraction (original magnification 50×). Second row: hematoxylin and eosin staining. Third row: staining for proliferation marker Ki-67 (red-brown nuclear stain, original magnification 200×). Fourth row: staining for the apoptosis marker TUNEL (brown, original magnification 200×). (**b**) FN16 and β-ABA inhibit the tumor growth. (**c**) FN16, AKBA, and β-ABA inhibit cancer cell proliferation. (**d**) FN16, AKBA, and β-ABA induce apoptosis in cancer xenografts. All data are mean ± SEM, *n* = 4. Comparison with control by Kruskal-Wallis one-way ANOVA on ranks and post-hoc by Dunn’s test with * *p* < 0.05, ** *p* < 0.01, and *** *p* < 0.001.

Treatment of cancer xenografts with FN16 reduced dose-dependently and significantly the tumor volume compared to the control group (Figure 6a,b). Also, treatment with β-ABA significantly reduced the tumor volume. AKBA-treated xenografts were only non-significantly smaller than control. Immunohistochemical analysis of the tumors revealed that FN16 had a dose-dependent inhibitory effect on cancer cell proliferation (Figure 6c). Also, AKBA and β-ABA significantly inhibited cancer cell proliferation. FN16, AKBA, and β-ABA all induced apoptosis in TNBC xenografts as shown by analysis of apoptosis by using the TUNEL technique (Figure 6d). Whilst the chemotherapeutic drug doxorubicin exhibited eminent cytotoxicity against MDA-MB-231 cells in vitro, in vivo its efficacy was not sufficient to induce a significant reduction of tumor size or to induce apoptosis during the treatment period. Notably, no systemic toxicity of the FN16 or BAs on the chick embryos was observed.

## 4. Conclusions

A comparative analysis of 16 frankincense nutraceuticals (FNs) revealed major differences in chemical compositions, cytokine modulatory properties, and cytotoxicities against triple-negative breast cancer cells. FNs with total boswellic and lupeolic acids (BAs and LAs) contents over 30% and/or β-ABA contents over 36 µg/mg significantly inhibited the expression of the proinflammatory cytokines TNF-α, IL-6, and IL-8. Moreover, FNs exhibited cytotoxic efficacy against triple-negative breast cancer cell lines MDA-MB-231, MDA-MB-453, and CAL-51 in vitro. In this regard, FNs with BAs and LAs contents over 30% and β-ABA contents over 50 µg/mg proved to be the most potent ones. A FN from this group and pure β-ABA inhibited growth and induced apoptosis in breast cancer xenografts in vivo. Moreover, a remarkable correlation between BAs and LAs contents, cytokine inhibition, and cytotoxicity against breast cancer cells could be observed. The contents of β-ABA exhibited the highest average correlation to inhibition of TNF-α, IL-6, and IL-8 cytokine release as well as cytotoxicity against breast cancer cells. Furthermore, pure β-ABA showed high cytotoxic efficacy against breast cancer cells in vitro and in vivo. Therefore, β-ABA should be considered as a compound for standardization of frankincense nutraceuticals and herbal preparations and it deserves further studies aiming at the development of new anticancer drugs.

## Figures and Tables

**Table 1:** Contents of boswellic and lupeolic acids in frankincense nutraceuticals (FN) quantified by HPLC-MS/MS analysis.

FN Sample	Concentrations of Boswellic and Lupeolic acids								
	Deacetylated Compounds (µg/mg)				Acetylated Compounds (µg/mg)				Σ (%) (*w/w*)
	KBA	LA	α-BA	β-BA	AKBA	ALA	α-ABA	β-ABA	
FN9	34.1	17.2	46.5	120.7	36.9	13.8	24.4	63.6	35.7
FN16	42.3	16.4	47.7	118.8	36.9	11.9	20.3	55.7	35.0
FN13	43.9	16.5	45.6	117.2	31.8	11.2	19.2	50.7	33.6
FN6	38.1	15.0	38.8	109.0	7.7	5.9	11.8	36.7	26.3
FN14	38.9	14.4	34.9	98.3	4.2	3.5	6.6	20.8	22.1
FN2	36.9	11.3	32.0	85.9	5.6	4.0	7.9	24.0	20.8
FN4	9.7	6.6	19.9	62.9	18.6	7.8	14.7	46.3	18.6
FN1	25.7	6.8	20.5	57.6	3.6	2.4	4.9	14.8	13.6
FN3	21.8	6.3	18.3	53.1	4.3	2.4	4.6	13.9	12.5
FN15	14.2	6.1	15.6	42.4	11.8	4.1	6.7	17.7	11.9
FN11	4.8	2.5	6.8	20.1	30.0	5.7	10.1	16.7	9.7
FN8	1.3	0.5	1.1	3.2	75.5	1.1	1.6	4.6	8.9
FN10	5.9	2.2	5.6	12.7	33.6	5.3	8.2	12.2	8.6
FN12	4.5	2.1	5.3	14.2	31.4	5.4	8.0	13.2	8.4
FN5	3.4	1.3	3.5	9.2	2.9	0.8	1.9	5.7	2.9
FN7	0.3	0.1	0.3	0.5	1.2	0.3	0.6	1.1	0.4

Samples are arranged by descending total contents of BAs and LAs (Σ in percent, *w/w*). KBA, 11-keto-β-boswellic acid; LA, lupeolic acid; α-BA, α-boswellic acid; β-BA, β-boswellic acid; AKBA, acetyl-11-keto-β-boswellic acid; ALA, acetyl-lupeolic acid; α-ABA, acetyl-α-boswellic acid; β-ABA, acetyl-β-boswellic acid.

**Table 2:** Correlation between BAs and LAs contents and inhibition of cytokine production by LPS-stimulated PBMC.

Compound	TNF-α		IL-1β		IL-6		IL-8		IL-10	
	*R*	*p*	*R*	*p*	*R*	*p*	*R*	*p*	*R*	*p*
KBA	−0.621	*	−0.241	> 0.05	−0.744	***	−0.665	**	−0.656	**
LA	−0.721	**	−0.341	> 0.05	−0.832	***	−0.791	***	−0.765	***
α-BA	−0.709	**	−0.356	> 0.05	−0.815	***	−0.774	***	−0.774	***
β-BA	−0.735	***	−0.359	> 0.05	−0.850	***	−0.815	***	−0.794	***
AKBA	−0.537	*	−0.714	**	−0.430	> 0.05	−0.530	*	−0.511	*
ALA	−0.867	***	−0.784	***	−0.884	***	−0.890	***	−0.918	***
α-ABA	−0.879	***	−0.797	***	−0.891	***	−0.897	***	−0.932	***
β-ABA	−0.847	***	−0.447	> 0.05	−0.932	***	−0.903	***	−0.900	***
Σ BAs and LAs	−0.753	***	−0.318	> 0.05	−0.835	***	−0.818	***	−0.785	***
TNF-α			0.612	*	0.959	***	0.968	***	0.944	***
IL-1β					0.535	*	0.600	*	0.624	**
IL-6							0.976	***	0.947	***
IL-8									0.947	***

Spearman’s rank correlation; R, correlation coefficient, * *p* < 0.05, ** *p* < 0.01, and *** *p* < 0.001, *n* = 16. PBMC, peripheral blood mononuclear cells.

**Table 3:** Cytotoxic efficacy of FNs against MDA-MB-231 cell line.

FN Sample	XTT: IC_50_ (µg/mL)		Regression: IC_50_ (µg/mL)		Absolute Residue (µg/mL)
	Mean	SEM	Value	SE	
FN16	6.0	0.9	6.7	0.9	0.7
FN13	6.6	0.6	5.7	0.8	1.0
FN9	7.0	0.4	7.5	1.0	0.5
FN4	9.5	0.9	8.4	0.9	1.1
FN6	11.0	1.8	9.2	1.1	1.8
FN14	11.6	0.7	13.7	0.9	2.0
FN2	12.2	1.6	13.1	0.7	0.9
FN15	13.2	1.1	15.4	0.7	2.2
FN12	13.2	0.6	14.5	0.8	1.3
FN11	13.9	0.5	13.6	0.8	0.3
FN10	15.0	0.9	14.4	0.9	0.6
FN1	19.4	2.7	18.1	0.8	1.3
FN3	19.5	1.0	18.4	0.8	1.1
FN8	21.5	3.6	21.4	1.5	0.2
FN7	30.7	6.7	30.4	1.4	0.3
FN5	44.3	8.5	/	/	/

XTT, IC_50_ values were determined experimentally by XTT assay (72 h, *n* = 3). Regression, IC_50_ values were determined by principal component regression (PCR). Data are mean ± standard error of the mean (SEM) or standard error of the regression model (SE). Sample FN5 as an outlier (Grubbs test) was excluded from the regression analysis. Absolute residue is the difference between experimentally determined values and those determined by regression analysis. Samples are arranged by cytotoxicity (XTT) in a descending order.

**Table 4:** Cytotoxic efficacy of FNs against triple-negative breast cancer cells (TNBC).

FN Sample	IC_50_ (µg/mL)			
	MDA-MB-453		Cal-51	
	Mean	SEM	Mean	SEM
FN9	14.5	0.9	3.8	0.5
FN13	12.9	0.6	3.9	0.6
FN16	17.3	0.5	4.0	0.3

XTT assay, 72 h, *n* = 3.

**Table 5:** Cytotoxic efficacies of the individual pure BAs and LAs against MDA-MB-231.

Compound	IC_50_			
	µg/mL		µM	
	Mean	SEM	Mean	SEM
KBA	12.0	0.4	25.4	0.9
LA	5.6	0.1	12.3	0.2
α-BA	5.0	0.4	10.8	0.9
β-BA	4.2	0.1	9.3	0.2
AKBA	3.4	0.1	6.6	0.2
ALA	13.4	0.5	26.9	1.0
α-ABA	3.6	0.5	7.2	1.0
β-ABA	2.9	0.4	5.9	0.8

XTT assay, 72 h, *n* = 3. Acetylated boswellic acids (AKBA, α-ABA, and β-ABA) are more potent compared to their deacetylated forms (KBA, α-BA, and β-BA). Differently, LA is more potent than ALA.

## Supplementary Materials

The following are available online at https://www.mdpi.com/2072-6643/11/10/2341/s1, Table S1: Frankincense nutraceutical (FN) capsule/pill contents and concentrations of boswellic and lupeolic acids per capsule/pill.; Table S2: Frankincense nutraceuticals (FNs) inhibit cytokine production by LPS-activated whole blood and isolated PBMC; Table S3: Boswellic and lupeolic acids inhibit cytokine production by LPS-activated PBMC; Figure S1: Correlations between contents of individual boswellic and lupeolic acids in frankincense nutraceuticals (FNs), FN cytokine inhibitory efficacies, and FN cytotoxicity against the MDA-MB-231 triple-negative breast cancer cell line (IC_50_); Figure S2: Preferential toxicity of FN1, AKBA, and β-ABA against MDA-MB-231 cells compared to PBMC. (**a**) FN1, (**b**) AKBA, and (**c**) β-ABA. XTT assay, 72 h.

## Appendix A

**Table A1:** Frankincense nutraceuticals (FNs) analyzed in the study.

FN Sample	Manufacturer/Distributor	Manufacturer’s Specifications			
		Product	Batch	Stated Content	RDA ^1^
FN1	Gall Pharma (Judenburg, Austria)	*Boswellia serrata* 200 mg GPH Kapseln	5260.53	200 mg *B. serrata* extract	2
FN2	Hecht Pharma (Bremervörde, Germany)	H15^®^ Weihrauch *Boswellia serrata* 350 mg	2260.52	350 mg *B. serrata* extract	1 × 1
FN3	Hecht Pharma (Bremervörde, Germany)	H15^®^ Weihrauch Kapseln 200 mg	5260.53	200 mg *B. serrata* extract	1 × 2
FN4	Heidelberg Pharmacy (Bisingen, Germany)	Weihrauch-Extrakt-Kapseln	HBMI07	300 mg *B. serrata* extract	3 × 1–2
FN5	The Nutri Store (Mauren, Liechtenstein)	*Boswellia carterii* Extrakt Kapseln	#7779529-1612	400 mg *B. carterii* extract	1 × 1
FN6	Wellnest Nutrazeutika (East Grinstead, England)	*Boswellia* Weihrauchextrakt	NB/BOS-1215189	400 mg *B. serrata* extract; 200 mg *B. carterii* extract	2
FN7	Vitabay (Masstricht, Netherlands)	Weihrauch Extrakt 400 mg	C17030104	400 mg *B. serrata* extract	2
FN8	Vitacost (Boca Raton, Florida)	Synergy 5-Loxin^®^ *Boswellia serrata* Extract	#703657	150 mg *B. serrata* extract	2
FN9	Schloss Pharmacy (Koblenz, Germany)	Indische Weihrauchkapseln	611142B	400 mg *B. serrata* extract	
FN10	Heilsteine Methusalem (Neu-Ulm, Germany)	*Sacra* Weihrauch Gold	WS/102017/NT	430 mg *B. sacra* gum resin	2 × 2
FN11	Heilsteine Methusalem (Neu-Ulm, Germany)	Weihrauch *Boswellia serrata*	WBS/2425/12AS	500 mg *B. serrata* gum resin	2 × 1
FN12	Olibanum B.V. (Kerkrade, Netherlands)	Boscari^®^, original afrikanischer Weihrauch	B0417	400 mg *B. carterii* gum resin	3 × 1
FN13	Biotikon (Gorxheimertal, Germany)	85 Premium *Boswellia serrata*	BOSW-200917	400 mg *B. serrata* extract	1
FN14	Gall Pharma (Judenburg, Austria)	*Boswellia serrata* Tabletten 400 mg	261.29	400 mg *B. serrata* extract	3 × 1
FN15	Gufic Biosciences Ltd. (Mumbai, India)	Sallaki^®^ Tablets 400 mg	AB17019	400 mg *B. serrata* extract	
FN16	Delphin Pharmacy (Langenau, Germany)	Weihrauch-Kapseln	BBD ^2^ 05.04.2018	400 mg *B. serrata* extract	

^1^ Recommended daily allowance; ^2^ Best-before date.
