# Supplementary material for: Comparative Investigation of Frankincense Nutraceuticals: Correlation of Boswellic and Lupeolic Acid Contents with Cytokine Release Inhibition and Toxicity against Triple-Negative Breast Cancer Cells
Source: Nutrients. 2019 Oct 2;11(10):2341. doi: 10.3390/nu11102341 (PMC6836131; doi:10.3390/nu11102341)
Supplement: Supplementary file 1 [file nutrients-11-02341-s001.pdf]

Supporting Information

Comparative Investigation of Frankincense Nutraceuticals: Correlation of Boswellic and Lupeolic Acid Contents with Cytokine Release Inhibition and Toxicity against Triple-Negative Breast Cancer Cells

Michael Schmiech [1], Sophia J. Lang [1], Judith Ulrich[1], Katharina Werner [1], Luay J. Rashan [2], Tatiana Syrovets [1] and Thomas Simmet [1,*]

[1] Institute of Pharmacology of Natural Products and Clinical Pharmacology, Ulm University, 89081 Ulm, Germany; michael.schmiech@uni-ulm.de (M.S.); sophia.lang@uni-ulm.de (S.J.L.); judith.ulrich@uni-ulm.de (J.U.); katharina.werner@uni-ulm.de (K.W.); tatiana.syrovets@uni-ulm.de (T.S.); thomas.simmet@uni-ulm.de (Th.S.)

[2] Medicinal Plants Division, Research Center, Dhofar University, Salalah 211, Oman; luayrashan@yahoo.com (L.J.R.)

[*] Correspondence: thomas.simmet@uni-ulm.de, Tel.: (+49) 731 500 62600; tatiana.syrovets@uni-ulm.de, Tel.: (+49) 731 500 62604

Table S1. Frankincense nutraceutical (FN) capsule/pill contents and concentrations of boswellic and lupeolic acids per capsule/pill.

FN Sample	Capsule/Pill Content [mg/unit]		Contents of Boswellic and Lupeolic Acids per Capsule/Pill [mg/unit]								
			Deacetylated Compounds				Acetylated Compounds				Total Content (Σ)
	Mean	SD	KBA	LA	α-BA	β-BA	AKBA	ALA	α-ABA	β-ABA	
FN1	502.1	14.2	12.9	3.4	10.3	28.9	1.8	1.2	2.4	7.4	68.3
FN2	503.1	4.4	18.6	5.7	16.1	43.2	2.8	2.0	4.0	12.1	104.5
FN3	489.8	13.9	10.6	3.1	8.9	25.9	2.1	1.2	2.2	6.8	60.9
FN4	336.4	15.9	3.2	2.2	6.7	21.1	6.3	2.6	5.0	15.6	62.7
FN5	584.3	6.3	2.0	0.7	2.0	5.4	1.7	0.5	1.1	3.3	16.8
FN6	567.1	7.6	21.6	8.5	22.0	61.8	4.4	3.3	6.7	20.8	149.1
FN7	809.8	5.0	0.2	0.1	0.3	0.4	0.9	0.2	0.5	0.9	3.5
FN8	346.8	14.9	0.4	0.2	0.4	1.1	26.2	0.4	0.6	1.7	30.9
FN9	380.7	12.4	13.0	6.5	17.7	45.9	14.1	5.3	9.3	24.2	135.9
FN10	415.0	6.8	2.4	0.9	2.3	5.3	14.0	2.2	3.4	5.0	35.5
FN11	375.4	0.9	1.8	1.0	2.5	7.6	11.3	2.1	3.8	6.3	36.3
FN12	418.0	9.1	1.9	0.9	2.2	5.9	13.1	2.3	3.3	5.5	35.1
FN13	390.7	8.7	17.2	6.4	17.8	45.8	12.4	4.4	7.5	19.8	131.3
FN14	700.9	5.6	27.3	10.1	24.4	68.9	2.9	2.4	4.6	14.5	155.2
FN15	659.6	8.7	9.3	4.0	10.3	28.0	7.8	2.7	4.4	11.6	78.2
FN16	449.4	1.2	19.0	7.4	21.4	53.4	16.6	5.3	9.1	25.0	157.3

FN capsule/pill contents ($n = 3$) and concentrations of boswellic and lupeolic acids per capsule/pill as analyzed by HPLC-MS/MS ($n = 3$ in duplicates). KBA, 11-keto-β-boswellic acid; LA, lupeolic acid; α-BA, α-boswellic acid; β-BA, β-boswellic acid; AKBA, acetyl-11-keto-β-boswellic acid; ALA, acetyl-lupeolic acid; α-ABA, acetyl-α-boswellic acid; β-ABA, acetyl-β-boswellic acid.

Table S2. Frankincense nutraceuticals (FNs) inhibit cytokine production by LPS-activated whole blood and isolated PBMC.

FN Sample	Whole Blood (*n* = 5)				PBMC (*n* = 7)									
	TNF-α [%]		IL-10 [%]		TNF-α [%]		IL-1β [%]		IL-6 [%]		IL-8 [%]		IL-10 [%]	
	Mean	SEM	Mean	SEM	Mean	SEM	Mean	SEM	Mean	SEM	Mean	SEM	Mean	SEM
FN1	88.3	8.7	80.2	16.4	112.2	37.7	122.5	27.2	104.1	26.4	93.0	13.7	57.6	16.6
FN2	87.9	5.6	92.3	11.3	43.7	21.6	120.1	29.6	19.9	11.7	26.8	9.3	28.4	12.8
FN3	55.7	10.9	89.1	17.9	137.6	49.6	141.6	22.4	93.5	24.9	84.6	11.7	47.0	11.4
FN4	90.3	5.7	90.4	9.6	14.4	7.2	90.8	38.4	7.2	3.3	14.4	4.8	21.6	11.4
FN5	93.0	8.1	80.6	13.0	179.8	49.0	92.8	17.1	133.1	24.0	114.5	13.3	85.0	21.6
FN6	76.1	7.9	68.9	21.4	22.1	12.5	68.3	19.9	11.6	6.5	17.6	6.7	26.4	14.6
FN7	92.3	10.2	77.6	14.4	207.1	57.2	171.3	49.7	246.4	88.6	178.5	62.2	86.1	23.9
FN8	92.0	9.5	67.4	5.1	101.7	35.7	100.9	22.3	112.1	42.7	70.9	17.2	63.4	19.2
FN9	75.0	5.6	70.3	8.0	22.5	13.3	59.8	15.4	8.1	4.6	13.5	4.1	24.5	13.6
FN10	79.2	6.4	77.0	6.7	61.5	23.8	58.3	14.1	57.6	21.5	50.9	11.1	30.0	12.0
FN11	81.7	8.0	80.3	8.6	60.6	20.3	87.4	32.0	46.1	14.4	47.3	12.0	27.4	11.0
FN12	80.2	7.0	62.4	12.1	106.5	38.4	71.2	13.3	85.7	33.8	67.4	19.1	55.6	24.3
FN13	85.4	7.7	81.9	9.9	18.2	9.6	82.5	28.6	6.7	3.1	16.5	4.4	24.9	13.6
FN14	91.8	9.3	93.4	3.6	67.3	18.3	122.4	37.0	35.0	14.3	45.9	14.4	32.5	13.4
FN15	56.7	14.3	80.7	20.4	76.1	24.9	98.2	38.2	49.5	16.7	61.0	14.7	35.5	15.4
FN16	97.6	9.7	74.8	16.8	26.1	11.9	58.5	21.9	12.4	8.1	22.2	10.8	24.3	12.3

Whole blood was pretreated with 30 µg/mL FNs (each) and PBMC with 10 µg/mL FNs (each) for 20 min and stimulated with LPS (10 ng/mL) for 18 h. Cytokine release was analyzed by ELISA and flow cytometry using CBA beads. LPS-stimulated control group without FN treatment = 100%, *n* = number of donors.

Table S3. Boswellic and lupeolic acids inhibit cytokine production by LPS-activated PBMC.

Compound	TNF-α [%]		IL-1β [%]		IL-6 [%]		IL-8 [%]		IL-10 [%]	
	Mean	SEM	Mean	SEM	Mean	SEM	Mean	SEM	Mean	SEM
KBA	101.3	7.2	71.5	18.0	94.0	26.0	106.3	30.7	96.1	20.9
LA	140.5	29.4	176.4	36.5	105.8	15.5	92.2	10.6	89.5	12.6
α-BA	114.8	26.2	128.7	16.5	96.0	19.3	88.9	12.5	113.0	22.0
β-BA	81.8	18.1	76.7	10.6	55.6	10.0	71.9	13.3	77.2	15.0
AKBA	53.5	17.9	44.8	14.3	61.6	19.4	60.8	15.9	86.0	25.9
ALA	88.8	26.4	91.1	15.1	84.3	24.1	79.5	15.8	92.3	20.2
α-ABA	84.6	15.2	84.5	15.0	76.8	14.8	79.1	9.5	104.2	25.1
β-ABA	65.0	15.6	62.6	11.5	59.3	15.1	64.1	10.3	101.3	26.4

PBMC were pretreated with BAs and LAs (3 μg/mL, each) for 20 min and stimulated with LPS (10 ng/mL) for 18 h; cytokine release was analyzed by flow cytometry using CBA beads. LPS-stimulated control group without compound treatment = 100%, n = 6 donors. KBA, 11-keto-β-boswellic acid; LA, lupeolic acid; α-BA, α-boswellic acid; β-BA, β-boswellic acid; AKBA, acetyl-11-keto-β-boswellic acid; ALA, acetyl-lupeolic acid; α-ABA, acetyl-α-boswellic acid; β-ABA, acetyl-β-boswellic acid.

	KBA	LA	α-BA	β-BA	AKBA	ALA	α-ABA	β-ABA	Σ BA/LA	TNF-α	IL-1β	IL-6	IL-8	IL-10	IC_{50}
**KBA**		0.941<br>2E-07	0.950<br>2E-07	0.924<br>2E-07	0.132<br>0.617	0.575<br>0.019	0.597<br>0.014	0.829<br>2E-07	0.909<br>2E-07	-0.621<br>0.010	-0.241<br>0.360	-0.744<br>0.001	-0.665<br>0.005	-0.656<br>0.006	-0.792<br>2E-07
**LA**			0.991<br>2E-07	0.991<br>2E-07	0.233<br>0.378	0.715<br>0.002	0.735<br>0.001	0.932<br>2E-07	0.976<br>2E-07	-0.721<br>0.001	-0.341<br>0.190	-0.832<br>2E-07	-0.791<br>2E-07	-0.765<br>0.000	-0.855<br>2E-07
**α-BA**				0.991<br>2E-07	0.240<br>0.360	0.715<br>0.002	0.735<br>0.001	0.932<br>2E-07	0.976<br>2E-07	-0.709<br>0.002	-0.356<br>0.171	-0.815<br>2E-07	-0.774<br>3.3E-05	-0.774<br>3.3E-05	-0.864<br>2E-07
**β-BA**					0.252<br>0.336	0.746<br>0.001	0.759<br>2.6E-04	0.956<br>2E-07	0.979<br>2E-07	-0.735<br>0.001	-0.359<br>0.167	-0.85<br>2E-07	-0.815<br>2E-07	-0.794<br>2E-07	-0.889<br>2E-07
**AKBA**						0.644<br>0.007	0.599<br>0.014	0.346<br>0.182	0.322<br>0.215	-0.537<br>0.031	-0.714<br>0.002	-0.430<br>0.094	-0.530<br>0.034	-0.511<br>0.042	-0.473<br>0.062
**ALA**							0.990<br>2E-07	0.849<br>2E-07	0.708<br>0.002	-0.867<br>2E-07	-0.784<br>2E-07	-0.884<br>2E-07	-0.890<br>2E-07	-0.918<br>2E-07	-0.897<br>2E-07
**α-ABA**								0.856<br>2E-07	0.715<br>0.002	-0.879<br>2E-07	-0.797<br>2E-07	-0.891<br>2E-07	-0.897<br>2E-07	-0.932<br>2E-07	-0.889<br>2E-07
**β-ABA**									0.935<br>2E-07	-0.847<br>2E-07	-0.447<br>0.080	-0.932<br>2E-07	-0.903<br>2E-07	-0.900<br>2E-07	-0.942<br>2E-07
**Σ BA/LA**										-0.753<br>3.7E-04	-0.318<br>0.224	-0.835<br>2E-07	-0.818<br>2E-07	-0.785<br>2E-07	-0.862<br>2E-07
**TNF-α**											0.612<br>0.012	0.959<br>2E-07	0.968<br>2E-07	0.944<br>2E-07	0.874<br>2E-07
**IL-1β**												0.535<br>0.032	0.600<br>0.014	0.624<br>0.010	0.561<br>0.023
**IL-6**													0.976<br>2E-07	0.947<br>2E-07	0.942<br>2E-07
**IL-8**														0.947<br>2E-07	0.920<br>2E-07
**IL-10**															0.898<br>2E-07

Centents: Correlation coefficient / *p*-value; $0.001 < p < 0.05$

Figure S1. Correlations between contents of individual boswellic and lupeolic acid in frankincense nutraceuticals (FNs), FN cytokine inhibitory efficacies, and FN cytotoxicity against the MDA-MB-231 triple-negative breast cancer cell line (IC_{50}). KBA, 11-keto-β-boswellic acid; LA, lupeolic acid; α-BA, α-boswellic acid; β-BA, β-boswellic acid; AKBA, acetyl-11-keto-β-boswellic acid; ALA, acetyl-lupeolic acid; α-ABA, acetyl-α-boswellic acid; β-ABA, acetyl-β-boswellic acid. Analysis of correlation was done by Spearman´s rank correlation (n = 16). Spearman´s correlation coefficients are shown as upper number, p values are colored according to the significance level and are shown beneath; red, no correlation.

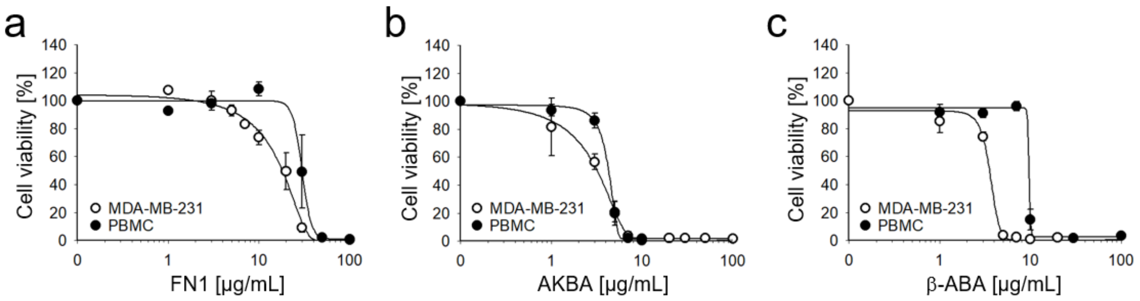

Figure S2. Preferential toxicity of FN1, AKBA, and β-ABA against MDA-MB-231 cells compared to PBMC. (**a**) FN1, (**b**) AKBA, and (**c**) β-ABA. XTT assay, 72 h, mean ± SEM, n = 3.
